# Unmet Healthcare Needs in People Living with Dementia: Exploring Associations with Sociodemographic and Clinical Characteristics ‐ Findings from the InDePendent Study’s Baseline Assessment

**DOI:** 10.1002/alz.087281

**Published:** 2025-01-09

**Authors:** Annelie Scharf, Fabian Kleinke, Bernhard Michalowsky, Anika Raedke, Stefanie Pfitzner, Franka Mühlichen, Maresa Buchholz, Neeltje van den Berg, Wolfgang Hoffmann

**Affiliations:** ^1^ German Center for Neurodegenerative Diseases e.V. (DZNE), Site Rostock/Greifswald, Greifswald Germany; ^2^ Institut for Community Medicine, University Medicine, University of Greifswald, Greifswald Germany

## Abstract

**Background:**

The healthcare needs of People living with Dementia (PlwD) (such as Alzheimer’s disease) are often unmet. Information about the needs of community‐dwelling PlwD and their association with sociodemographic and clinical characteristics is needed to fill the knowledge gap regarding factors influencing unmet needs among PlwD and to conduct a comprehensive needs assessment to develop tailored interventions.

**Method:**

We analyzed baseline data of the multi‐centre cluster‐randomized controlled trial (InDePendent) using descriptive statistics to describe patients' sociodemographic and clinical characteristics and Poisson regression models to predict unmet needs, separated by sex. Data were collected personally via face‐to‐face interviews.

**Result:**

Most of the n = 417 participating PlwD were mild to moderately cognitively impaired, were not depressed, had an average of 10.8 diagnoses, took 6.7 medications, and had, on average, 2.4 unmet needs (62% of PlwD had at least one unmet need) measured by the Camberwell Assessment of Need for the Elderly (CANE). Low social support, a high body‐mass index, a lower education, functional impairment, and worse health status were associated with more unmet needs, regardless of sex. More depressive symptoms, a poor financial situation, and living alone increased unmet needs in women, while being recently treated by a general practitioner decreased unmet needs. The number of medications increased unmet needs in male PlwD.

**Conclusion:**

PlwD had a broad array of unmet healthcare needs, indicating primary healthcare provision improvement potentials. The results underscore the significance of early assessment of patient’s clinical characteristics and unmet needs as a basis for individualized gender‐sensible intervention strategies.

